# The Role of Exosomes in Lysosomal Storage Disorders

**DOI:** 10.3390/biom11040576

**Published:** 2021-04-15

**Authors:** Adenrele M. Gleason, Elizabeth G. Woo, Cindy McKinney, Ellen Sidransky

**Affiliations:** Medical Genetics Branch, National Human Genome Research Institute, National Institutes of Health, Bethesda, MD 20892, USA; adenrele.gleason@nih.gov (A.M.G.); elizabeth.woo@nih.gov (E.G.W.); dr.cmckinney@gmail.com (C.M.)

**Keywords:** exosomes, endocytic pathways, neurodegenerative disease, Gaucher disease, Parkinson disease, lysosomes, lysosomal storage disorder

## Abstract

Exosomes, small membrane-bound organelles formed from endosomal membranes, represent a heterogenous source of biological and pathological biomarkers capturing the metabolic status of a cell. Exosomal cargo, including lipids, proteins, mRNAs, and miRNAs, can either act as inter-cellular messengers or are shuttled for autophagic/lysosomal degradation. Most cell types in the central nervous system (CNS) release exosomes, which serve as long and short distance communicators between neurons, astrocytes, oligodendrocytes, and microglia. Lysosomal storage disorders are diseases characterized by the accumulation of partially or undigested cellular waste. The exosomal content in these diseases is intrinsic to each individual disorder. Emerging research indicates that lysosomal dysfunction enhances exocytosis, and hence, in lysosomal disorders, exosomal secretion may play a role in disease pathogenesis. Furthermore, the unique properties of exosomes and their ability to carry cargo between adjacent cells and organs, and across the blood–brain barrier, make them attractive candidates for use as therapeutic delivery vehicles. Thus, understanding exosomal content and function may have utility in the treatment of specific lysosomal storage disorders. Since lysosomal dysfunction and the deficiency of at least one lysosomal enzyme, glucocerebrosidase, is associated with the development of parkinsonism, the study and use of exosomes may contribute to an improved understanding of Parkinson disease, potentially leading to new therapeutics.

## 1. Introduction

Cells have several different means of conveying intercellular bioinformation. This may occur by direct cell-to-cell contact, through signaling via secreted soluble molecules, and by small vesicle-packaged molecules [1]. These different **vesicles (see Appendix A for the definitions of terms shown in bold)** include **exosomes**, **ectosomes**, **apoptotic bodies,** and **autophagosomes**. Exosomes, the subject of this review, [2,3] were initially described as waste material, encapsulated in small lipid vesicles, captured from the cytoplasm of maturing reticulocytes. However, it subsequently became clear that exosome cargos represent a heterogeneous source of normal or pathological biomarkers that capture a cell’s metabolic state at a given point in time. Exosomes are formed from endosomal membranes and their evolution intersects with steps in **autophagy-lysosomal pathways (ALPs)**. As they mature, exosomes become filled with a selected set of sorted biomolecules. These may include lipids, metabolites, **mRNAs, miRNAs,** and proteins. While many important aspects of exosome biology in health and disease remain to be defined, **exosomal cargos** have at least two fates. They may act as intercellular messengers (e.g., paracrine signaling) and/or inter-organ communicators (e.g., metastatic disease). They also carry defined intracellular cargo from interconnected autophagic/lysosomal degradation pathways in the cell. Depending on the cell of origin and the cargo source, the exosomes contribute to homeostatic or metabolic regulation, as well as to disease processes, including viral infection, cancer metastasis, and neurodegenerative diseases [4]. While a better understanding of the role of exosomes in normal physiology is just now emerging, their role in pathological conditions, such as neurodegeneration, has been more rigorously examined.

## 2. The Biogenesis of Exosomes: A Subclass of Extracellular Vesicles (EVs)

**Figure 1:**
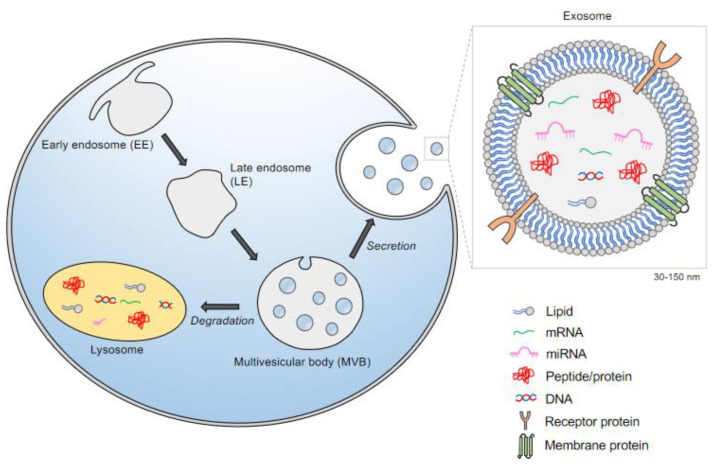
Intracellular biogenesis of exosomes. Exosomes can be formed through the endocytic trafficking pathway. These vesicles are generated by limited inward budding of the late endosome to form the intraluminal vesicles (ILVs). ILVs are components of multivesicular bodies (MVB). Two routes can occur at the MVB: 1. Degradation, resulting from fusion with lysosomes and 2. Secretion, that takes place when multivesicular bodies (MVBs) fuse with the plasma membrane and release their contents into the extracellular space. Secreted exosomes can be captured by nearby or distant cells and regulate the physiological state of the recipient cell.

All cells release exosomes, 30–150 nm membrane-bound vesicles that derive from the invagination of endosomal membranes. Exosomes acquire their content both from biosynthetic routes and by **endocytosis**, a form of intracellular trafficking. Endocytosis begins with the invagination of the plasma membrane (PM), which can be **clathrin-coated**. Upon internalization, these vesicles first fuse with the **early endosome** (EE), also described as the sorting endosome. Here, major decisions on the next trafficking route occur. Classically, cargo destined for degradation enter the endosomal-lysosomal system, where the EEs mature and begin to invaginate parts of their membranes. These **intraluminal vesicles** (ILVs) then become part of the **multivesicular bodies** (MVB), organelles comprised of vacuoles delimited by a single membrane [5]. The MVBs host multiple vesicles, and depending on the invagination size, these vesicles range from 50-150 nm in diameter [6]. Two trafficking routes can occur at the MVB. Membrane components and other macromolecules encapsulated in these vesicles can be hydrolyzed after fusing with lysosomes. Enzymes within the lysosomes then degrade the delivered carbohydrates, proteins, fats, and other cellular products into smaller and simpler components that are then recycled as building blocks for new molecules (Figure 1). Alternatively, these vesicles become future exosomes when released from cells, through a process called secretion. In this trafficking step, MVBs fuse with the plasma membrane and release the ILVs as exosomes [7]. The exosome membrane composition is similar to that of the plasma membrane and contains inserted and captured cytoplasmic proteins. The internal cargo within small vesicles is sorted, sometimes as part of protein complexes that assist in moving and/or sorting. This complex of proteins is also known as **endosomal sorting complex required for transport (ESCRT)**-associated pathways [8].

## 3. Exosome Cargo and Transport:

The recent explosion of exosome research has been driven by their newly defined role in intercellular vesicular trafficking. This results in the transmission of their signaling cargo by means of secretion at the plasma membrane into the extracellular space, enabling uptake in recipient cells. Characterizing the content of exocargo can provide information about exosome biogenesis, potential effects on the recipient cell, and insights into disease diagnosis, progression, and prognosis, by serving as biomarkers. Exocargo may also be useful in monitoring cellular responses to disease treatments [9]. Depending on the cell of origin, exosomes may contain cargos enriched in proteins (such as TSG101 and Alix) [10,11], miRNAs [12], translatable mRNAs [13], lipids (i.e., ceramide) [14,15], and other bioactive molecules (see Exocarta.org [16] a database of biomolecules reported in exosomes). Surprisingly, the propagation and release of exosomes is conserved across species spanning from fungi to humans, and thus, it is possible that exosomes from other species could communicate signals to humans, e.g., bovine exosomes in milk. Horizontal transfer between species, or between cells of multiple organs in the body, could have either a beneficial or detrimental effect. The exact role that these exoproteins and macromolecules play in external cellular communication is currently under investigation [17,18].

Our knowledge of how the selection and packaging of cargo into exosomes is accomplished is advancing, although many questions remain unresolved. The cargo and membrane content of exosomes differs depending on the on the nature of the parent cell. This suggests that there are mechanisms in the cell directing the sorting of specific molecules [19] and enzymes [20,21], indicating that active processing helps define the content of mature exosomes. Several complexes appear to be involved in sorting and packaging pathways, including Rab family members, p53 and its effector tumor suppressor-activated pathway (TSAP) [22,23,24]. In the case of miRNA content, it was shown that the internal sequence GGAG at the 3′-end can direct certain miRNAs to ILVs. Similarly, in murine hepatocytes, the GGCU motif located on the 3′-end of miRNAs is recruited to the RNA binding protein SYNCRIP (synaptotagmin-binding cytoplasmic RNA-interacting protein) [25]. This sequence specific interaction directs miRNA sorting into exosomes [25]. Further studies are needed to improve our understanding of how miRNAs are sorted into these membrane-bound exosomes [1]. A better characterization of the cargoes intrinsic to exosomes derived from different cell types will help to clarify the molecular mechanisms governing exosome function and their role in various aspects of biology and disease.

## 4. Exosomal Signatures: Lessons from the CNS

Most cell types in the central nervous system (CNS) release exosomes, including astrocytes, microglia, oligodendrocytes, and neurons. As potential vectors for intercellular communication, exosomes harbor distinct molecular contents that reflect their donor cell. This section will summarize the signature of exosomes found in the cell types comprising the CNS. This is particularly relevant to specific lysosomal storage disorders, as most have CNS involvement that is only partially understood.

### 4.1. Exosomes from Astrocytes

It is well established that **astrocytes** influence neuronal function, however the molecular mediators that regulate this process continue to be under intense investigation. To study their effects on neural uptake, differentiation, and maturation, astrocyte-derived extracellular vesicles (ADEVs) were isolated from human primary astrocytes in culture [26]. In this study, the proteomic signature of exosomes derived from astrocytes revealed astrocyte-specific markers including GFAP, excitatory amino acid transporter 1 (SLC1A3/GLAST), and glucose transporter member 1 (SLCA1/GLUT1). In addition, the authors also examined the exocargo of astrocytes treated with the classic pro-inflammatory cytokine interleukin-1 β, termed IL-1β-ADEVs. When compared to controls, the proteomic profile was enriched in proteins characteristic of reactive astrocytes [26]. To examine the physiological consequences of neurons treated with IL-1β-ADEVs, different assays were performed, showing delayed outgrowth and a reduction in neurite length, total surface area, node number, and neuronal firing [26].

Other ex-vivo studies showed that exosomes purified from astrocyte processes freshly prepared from adult rat cerebral cortex participated in signal transmission and could target both near and distant sites. The exosomes were enriched for markers typically found on astrocytes, including GFAP and Ezrin [27], as well as the neuroprotectant protein neuroglobulin (NGB). The released exosomes selectively targeted neurons, where they were then internalized, suggesting that exosomes could deliver NGB originating from astrocytes to neurons. It was also observed that in astrocytes, apoptosis is associated with the release of PAR-4/ceramide-containing lipid exosomes [27].

### 4.2. Exosomes from Cortical Neurons

Exosomes are also secreted from **cortical neurons**, with important consequences. In one study, the biochemical composition of exosomes prepared from the cultured medium of primary cultures of rat cortical neurons at embryonic day 16 was evaluated using mass spectrometry. Proteomic profiling identified an enrichment of integral membrane protein GluR2/3, the α-amino-3-hydroxy-5-methyl-4-isozazolepropionic acid receptors (AMPARs), and the specific cell adhesion molecule L1 [28]. Interestingly, this analysis did not show enrichment of NR1 subunits of the N-methyl-D-Aspartic Acid Receptor 1 (NMDA) glutamate receptor, suggesting that GluR2/3 secretion is specifically facilitated through exosomes. In contrast, these exosomes did not display the plasma membrane protein, NA^+^/K^+^-ATPase, providing evidence that the exosome fractions were not contaminated with plasma membrane [28].

Further studies showed that mature cortical neurons from the somato-dendritic compartment are capable of releasing exosomes [29]. Here, they showed that the release of exosomes from 15 day cortical and hippocampal neuronal cultures was regulated by calcium influx and glutamatergic synaptic activity. When treated with ibicucullin or pirocotoxin, antagonists of GABA receptors known to enhance glutamatergic spontaneous activity, a mass release of exosomes was detected [29]. As a proof of principle, the subsequent addition of either an AMPAR or NMDA antagonist attenuated the GABA_A_-induced exosomal secretion. Taken together, these experiments demonstrate that secretion of exosomes is regulated by glutamatergic activity in cortical neurons.

Furthermore, exosomes secreted by cortical neurons upon glutamatergic synapse activation were selectively endocytosed by neurons, whereas the exosomes derived from neuroblastoma cells were taken up by glial and neuronal cells without bias [30]. Thus, the uptake of exosomes is somehow specified by the parental cell, suggesting a novel mechanism of inter-neuronal communication.

### 4.3. Exosomes from Oligodendrocytes

**Oligodendrocytes** insulate axons with a multilayered myelin sheath. It has been proposed that exosomes secreted from oligodendrocytes may relay molecular cues that support glia-mediated trophic nutrients to axons [31]. In one study, myelinating oligodendrocytes were found to secrete exosomes in a Ca^2+^-dependent manner [32]. This observation supports other studies suggesting that exosome release is triggered by neuronal activation [29]. In another study, Frühebeis et al. showed that in contact-independent cocultures of neurons and oligodendrocytes, the neurotransmitter glutamate was able to trigger the secretion of oligodendroglial exosomes [33]. This release was mediated by Ca^2+^ entry through oligodendrogial NBQX (2,3-dihydroxy-6-nitro-7-sulfamoyl-benzo[f]quinoxaline-2,3-dione); NMDA and AMPA receptors [33]. Furthermore, exposure of neurons to oligodendroglial-derived exosomes increased the action potential firing rate, altering both the transcriptome and cellular signal transduction pathways [34]. These exosomes also transferred protective proteins, including catalase and superoxide dismutase (SOD) [34]. Lipid extracts from exosomes isolated from primary cultured oligodendrocytes were analyzed by thin layer chromatography (TLC). These studies identified an enrichment of the canonical myelin lipids galactocerebroside and sulfatide [32]. Proteomic profiling demonstrated that a fraction of these exosomes contained canonical myelin proteins, including myelin proteolipid protein (PLP), 2′3′-cyclic-nucleotide-phosophdiesterase (CNP), myelin oligodendrocyte glycoprotein (MOG), and, to a lesser extent, myelin basic protein (MBP) [32]. Further classification of the proteins identified by mass spectroscopy revealed a spectrum of diverse protein families, including those involved in signal transduction pathways, lipid metabolism, oxidative stress, cellular metabolism, and intriguingly, nuclear proteins [32].

## 5. Studies of Exosomes in Specific Lysosome Storage Disorders (LSDs)

Lysosomal storage diseases (LSD) include over 70 heritable inborn errors of metabolism characterized by lysosomal dysfunction. Mutations in the genes encoding lysosomal proteins lead to substrate accumulation within the lysosome, which can result in cell dysfunction and cell death [35]. LSDs are individually rare, but as a group have an estimated incidence of around 1 in 5000 [36]. These genetically and clinically heterogeneous disorders affect multiple systems, often manifesting with neurological involvement and neurodegeneration.

LSDs result from defects in lysosomal proteins and enzymes, but also endocytic trafficking proteins, integral membrane proteins, and lipids, as well as regulatory proteins [35,37,38]. Collectively, these disorders can be further divided into subcategories based upon the stored materials, such as mucopolysaccharidoses, glycoproteinoses, and lipidoses [38]. The composition of the accumulated materials is intrinsic to each LSD. It has been proposed that in an effort to alleviate the buildup, these undigested materials are, in part, redirected into the extracellular space by lysosomal exocytosis [39]. In the following section, we will review studies of the release of exosomes/EVs in six specific LSD models (Table 1).

### 5.1. Metachromatic Leukodystrophy

Metachromatic leukodystrophy (MLD) is an LSD caused by autosomal recessive mutations in *ARSA*, the gene coding for lysosomal hydrolase arylsulfatase A (ASA) [40]. This enzyme is responsible for the breakdown of the spingolipid-3-*O*-sulfogalactosylceramide (sulfatide) in oligodendrocytes and distal tubule kidney cells [41]. Patients deficient in this enzyme have increased sulfatides in their urine and cerebrospinal fluid. The buildup of sulfatides damages cells of the CNS, in part by affecting the myelin sheath, as well as the nerve fibers protected by this sheath, resulting in weakness and neurodegeneration. Studies have suggested that the elevated sulfatides present in the urine is a cellular response to dying tubular kidney cells [41]. In an effort to investigate the underlying molecular mechanisms, primary cell cultures of kidney tubule cells from ASA deficient mice were established, demonstrating calcium-induced lysosomal exocytosis [42]. Examination of the content secreted into the cell culture medium showed sulfatides in ASA-deficient cells but not in controls. This study is one of the first to propose that cells are eliminating sulfatides through lysosomal exocytosis. Taken together, this observation provides a potential mechanism for the presence of sulfatides in the biological fluids of patients with MLD.

### 5.2. Mucolipidosis Type IV

The autosomal recessive LSD Mucolipidosis type IV (MLIV) results in severe neurological and ophthalmologic impairment, motor delay, and gastric dysfunction [43,44]. MLIV is caused by pathologic variants in *MCOLN1,* a member of the transient receptor potential (TRPML1) cation channel gene family [44]. This gene encodes the protein, mucolipin-1 (MLN1), the master Ca^2+^ release channel in the lysosome. However, how the lack of MLIV leads to neurodegeneration is largely unknown. Similar to studies in MLD [42], fibroblasts derived from patients with MLIV also exhibited dramatic impairment in lysosomal exocytosis [45]. This was evaluated by assays measuring the cleavage product of the soluble lysosomal enzyme N-acetyl-β-D-glucosaminidase (NAG). These studies revealed that the amount of NAG released from cultured control fibroblast cell lines was 250-fold higher than the amount released in lines from patients with MLIV [45]. Subcellular localization studies revealed that MLN1 translocates to the plasma membrane and correlates with the percentage of lysosomal organelles available for exocytosis. Transfecting different patient cell lines with wildtype *MLN1* corrected impaired lysosomal exocytosis and restored the channel activity [45]. This work identified the first ion channel, MLN1, found to play a role in lysosomal exocytosis.

Additional studies further examined the role of MLN1 and exosomal release. Depletion of MLN1 from mature adipocytes reduced the exosomal markers CD9, CD81, CD63, and Hsp70 [46]. Notably, isolated plasma membrane from mature adipocytes showed that loss of MLN1 led to a 50% reduction in levels of the lysosomal marker LAMP-1. LAMP-1 is enriched at the plasma membrane when cells are undergoing exocytosis [46]. Taken together, these results indicate that MLN1 plays a role in the trafficking of lysosomes to the plasma membrane, as well as in lysosomal mediated-exosomal secretion.

### 5.3. Sialidosis

Pathogenic gene variants in *NEU1* disrupt N-acetyl-α-neuraminidase, the lysosomal enzyme that initiates the catabolism of sialyl-glycoconjugates by removing terminal sialic acid residues [47,48]. Perturbations in this pathway cause the LSD sialidosis (also known as ML1) in humans. While there are different types of sialidosis, with differing degrees of clinical severity, they all affect the CNS. Patients with sialidosis have been shown to have diffuse brain atrophy.

Complementary studies, conducted in both murine *neu1^−/−^* macrophages and fibroblasts from patients with type II sialidosis [49] showed that cell lines deficient in this lysosomal enzyme had a subcellular redistribution of LAMP-1, a resident lysosomal marker, to the plasma membrane. Supportive experiments using electron microscopy showed that macrophages from a *neu1^−/−^* mouse were enriched with “clusters” of lysosomes proximal to the PM, reflecting excessive lysosomal exocytosis. Subsequent studies described the impact of human NEU1 deficiency on exosome release in patient fibroblasts, again showing increased levels of oversialyated LAMP-1 at the PM was accompanied by increased exocytosis.

Functional studies were used to examine the molecular consequences of exosomes released by *neu1^−/−^* mice in muscle and connective tissue [50]. Murine *neu1^-/ -^* fibroblasts exhibited excessive release of exosomes carrying profibrotic signaling molecules, including activated transforming growth factor-β (TGFβ) and wingless-related integration site (WNT)/ β-catenin signaling ligands. These signaling molecules are known to propagate fibrotic signals and trigger myofibroblast transdifferentiation [51,52]. Adding exosomes derived from *neu1^−/−^* myofibroblasts into the cultured medium of normal fibroblasts converted them into myofibroblasts [50]. However, when wild-type N-acetyl-α-neuraminidase activity was restored in the *neu1^−/−^* murine myofibroblasts, exosomes isolated from the rescued cell line no longer converted normal fibroblasts into myofibroblasts, suggesting that restoring N-acetyl-α-neuraminidase activity corrected the excessive release of exosomes carrying profibrotic signaling molecules [50].

### 5.4. Cystinosis

Cystinosis is a lysosomal storage disease caused by pathogenic variants in the gene *CTNS,* which encodes a lysosomal membrane cystine transporter, cystinosin [53]. This multi-systemic disorder leads to the accumulation of lysosomal cystine and is the main cause of hereditary renal Fanconi syndrome [53]. The addition of microvesicles and/or exosomes derived from both mesenchymal stem cells (MSCs) and transduced insect cells containing wildtype *CTNS* protein to fibroblasts from patients with cystinosis corrected the cystine levels [54,55]. Additional reports have identified **tunneling nanotubules** (TNT) as an alternative endocytic trafficking mechanism that can mediate the cystine clearance. Co-culture assays showed that TNTs produced by wildtype macrophages allowed for the transfer of cystinosin-bearing lysosomes into the cystinosin-deficient fibroblasts [54]. Conversely, cystinotic fibroblasts used the same TNTs to unload aberrant cystine-filled lysosomes into wildtype macrophages. In fact, TNT corrected cystine levels with greater efficiency than secreted exosomes/microvesicles from wildtype MSC or macrophages. Taken together, these observations showed that bidirectional exchange of vesicles and cargo between cell types can occur through TNTs. This work also demonstrated that vesicles released from stem cells are capable of delivering the correct cargo to repair deficits in a neighboring cell.

### 5.5. Niemann-Pick Type C

Niemann–Pick type C (NPC) disease is caused by bi-allelic variants in two genes, *NPC1* and *NPC2.* Loss of *NPC1* causes abnormal intra-lysosomal accumulation of unesterified cholesterol and sphingolipids in the brain, liver, and spleen [56]. While the age at presentation and the initial manifestations are variable, progressive dementia and neurological signs are almost universal among patients with NPC.

To examine whether *NPC1* impacted exosome secretion, Strauss et al. (2010) showed that both fibroblasts from patients harboring pathogenic *NPC1* variants, as well as siRNA-mediated knockdown of *NPC1* in wild-type oligodendrocytes showed elevated exosomal cholesterol release [57]. Then, oligodendroglial cells were challenged, either by increasing amounts of free cholesterol in the media, or using an inhibitor, U18666A, known to sequester cholesterol into the late endosome/lysosome compartments [57]. In both scenarios, treated cells had a striking increase in exosome secretion. Interestingly, the release of exosomal cholesterol was dependent on the function of the known exosome marker flotillin. Collectively these are the first observations reporting an increase in exosomal cholesterol secretion in cell lines that disrupt *NPC1* function. Taken together, the secretion of exosomal cholesterol in response to lysosomal dysfunction may represent an additional mechanism orchestrating intracellular cholesterol homeostasis. This first study documenting the levels of exosomes in an LSD, requires further validation in in vivo models.

### 5.6. GaucherDdisease

Gaucher disease (GD), resulting from deficient lysosomal glucocerebrosidase, is caused by bi-allelic variants in *GBA1* and is the most common genetic risk factor for the more common neurodegenerative disorder, Parkinson disease (PD). Deficiencies in this lysosomal enzyme disrupt the conversion of glucosylceramide into glucose and ceramide, resulting in the accumulation of glucosylceramide, as well as its deacylated form glucosylsphingosine, in lysosomes. There are both neuronopathic and non-neuronopathic forms of GD, although the basis for the neuropathology seen is not fully understood.

Recent studies performed using patient plasma indicated that lysosomal dysfunction in patients with GD leads to a striking alteration of plasma exosomal size and morphology [58]. **Dynamic light scattering** (DLS) detected three distinct populations of EVs, finding that the proportion of exosomes around 100 nm in diameter was significantly increased in the GD cohort. Complementary studies using cryo-EM showed an abundance of vesicles with altered morphology in samples from patients with GD. High resolution images of EVs isolated from these patients exhibited multilayered vesicles of larger size, when compared to controls [58]. In contrast to the other LSDs described in this section, the concentration of EVs released did not significantly differ between patients with GD and controls.

### 5.7. Lysosomal Storage Disorders in General

The focal point of these studies converges on the notion that perturbations in lysosomal function influence lysosomal exocytosis and therefore EV secretion (Table 1). Several studies have demonstrated that lysosomal dysfunction enhances EV secretion by upregulating exocytosis. It has been postulated that elevated EV secretion serves to relieve the accumulation of byproducts in the lysosome. Other studies examining exosome secretion in patients with GD reported an altered morphology when compared to controls, although the total exosome concentration released was similar [58]. As described previously, the accumulation of metabolites and partially undigested cellular materials is specific to each LSD. However, two unifying observations are commonly found in these disorders: 1. increased lysosomal exocytosis, and 2. elevated EV secretion. It is thought that both mechanisms serve as complementary routes to remove the buildup of unwanted waste in the lysosome [39]. Furthermore, it has been suggested that the accumulated byproducts in the lysosome may be shunted to the ILVs found in MVBs. These ILVs, containing unwanted products from the lysosomes, become exosomes when the MVBs fuse with the plasma membrane [39]. These are attractive hypotheses; however crucial unanswered questions remain. The molecular mechanisms detailing the coordinated release of exosomes, whether via the redirection of MVBs and/or lysosomes to the plasma membrane are still unknown. Future work examining the role of exosomes in various LSDs could provide a model system to explore, not only the molecular mechanisms that govern exosome secretion, but also their role in cellular homeostasis.

## 6. Exosomes as Therapeutic Agents for Treating Lysosomal Storage Disorders

During the past few decades, our ability to treat many of the LSDs has greatly improved. Current treatments include enzyme replacement therapy (ERT) and substrate reduction therapy (SRT), while pharmacological chaperones and gene therapy [59] are emerging options. One challenge in treatment is our current limited ability to deliver therapies across the blood–brain barrier. Extracellular vehicles (EVs) and exosomes could help to address this challenge by serving as drug-delivery vehicles. Since these vesicles can carry cargo between adjacent cells and organs, and efficiently cross the blood–brain barrier [59], they are attractive candidates for drug development. [60].

One LSD for which exosome-based therapy is being explored is Gaucher disease, where preliminary work reported the potential of opto-genetically engineered exosomes for treating GD [61]. A 2019 study described a method of producing enzyme-loaded HEK293-derived exosomes for the targeted delivery of glucocerebrosidase to endocytic compartments of recipient cells [62]. The authors fused glucocerebrosidase to exosomes, targeting the transmembrane protein vesicular stomatitis virus glycoprotein (VSVG), and loaded these fusion proteins onto exosomes. The exosomes were taken up by recipient cells, which showed significantly enhanced gluocerebrosidase activity. They designed exosomes that presented functional enzymes in two different spatial conformations, finding no significant differences [62]. While this approach demonstrated potential, it should be further confirmed using patient-derived or animal models of GD.

The application of EVs for treatment has also been explored for ceroid lipo-fuscinoses 2 (CLN2) or Batten disease. CLN2 is a lysosomal storage disorder that results from dysfunctional lysosomal enzyme tripeptidyl peptidase-1 (TPP1), encoded by *TPP1*. A 2019 study used macrophage-derived EVs to deliver soluble TPP1 protein for the treatment of CLN2 [63]. In one approach, they transfected macrophages with the *TPP1*-encoding plasmid DNA. In another, they loaded naïve EVs with TPP1 therapeutic enzyme ex vitro, using sonication or saponin permeabilization. Both techniques successfully incorporated enzymatically active TPP1 into EVs, which then delivered TPP1 to target cells in an in vitro model of CLN2. Around 70% of the enzyme was delivered to lysosomes, the target organelles. Using a late-infantile neuronal ceroid lipofuscinosis mouse model, they found that the EVs were delivered to the brain, and noted an increased lifespan after intraperitoneal administration of EV-TPP1. This study further supports the potential use of macrophage-derived EVs as drug delivery vehicles for the treatment of LSDs.

Exosome-based strategies are also under investigation for the treatment of NPC. The NPC protein is a large transmembrane cholesterol transporter and these characteristics limit the applicability of an ERT-type or gene therapy approach. Engineered exosomes are currently being developed to carry a functional copy of the NPC protein to neural cells [64].

While exosome therapeutics for LSDs is still an emerging field, recent and ongoing work is promising. As with exosome therapy applied to any other disease, there are still challenges to address before these strategies can be widely implemented. These include determining the exosome’s loading capacity and the half-life of the cargo, exploring exosome dosage and biodistribution, and evaluating the kinetics of exosome uptake at the target cells [59]. A better understanding of exosomes, as well as further investigation into their in vivo limitations and any potential immunological response is necessary for the successful development of such therapeutic strategies.

## 7. Concluding Remarks

This review highlighted the biogenesis and signature of exosomes, their role as intercellular messengers in neuronal circuitry, their fate in a subset of LSDs, and explored the potential applications of exosomes in cell-based therapeutics to treat LSDs. Treatments targeting the nervous system are a crucial unmet need in the LSDs. Further research is also needed to determine both the role and predictability of exosomal cargo as biomarkers for disease states. While the landscape of exosome research continues to evolve, major questions and challenges remain. In particular, in vivo studies capturing the exosomal signature specific to each cell type in the CNS are crucial for interpreting their physiological functions. Such studies will allow us to examine how substrate accumulation or lysosomal dysfunction in the LSDs may influence the spatiotemporal properties of EVs and their impact on the neuronal circuitry in vivo. Further technological advances are required to improve exosomal purification, and improved molecular markers are needed to definitively separate and classify each subpopulation.

A major advantage of the EV transport network over other cellular communication networks (paracrine, autocrine, exocrine) is that EVs can deliver their membrane encapsulated effector cargos across inter-organ distances without becoming diluted or degraded. However, the mechanisms that target a specific subset of EVs to a particular cell type or tissue remain to be explored. While this field is rapidly evolving, further rigorous basic science investigations are needed to better guide the clinical use of exosomes in targeted therapies and diagnostics.

## Figures and Tables

**Table 1:** Selected Lysosomal Storage Disorders (LSD).

LSD	Metabolite Accumulation	Consequence
Metachromatic leukodystrophy	Sulfatide (spingolipid-3-O-sulfogalactosylceramide)	Increased lysosomal exocytosis
Mucolipidosis type IV	Phospholipids, gangliosides	Increased lysosomal exocytosis
Sialidosis	Sialylated oligosaccharides and glycopeptides	Increased lysosomal exocytosis Identified NEU1 as a negative regulator in lysosomal exocytosis
Cystinosis	Cystine	Microvesicles/exosomes containing wildtype CTNS protein can correct
Niemann-Pick type C	Cholesterol	Increased exosomal-cholesterol secretion in vitro
Gaucher disease	Glucosylceramide, glucosylsphingosine	Increased number of exosomes, aberrant morphology

## Data Availability

N/A.

## Appendix A

**Vesicles**

Enclosed by a lipid bilayer, these tiny spherical sacks are filled with fluid from the parental cell. Depending on their origin, they can carry out a variety of functions including transport and signal transduction.

**Extracellular vesicles (EVs)**

These lipid bilayer vesicles are naturally released from the cell and range in diameter (50–5000 nm) [65]. Depending on their biogenesis and release pathways, these vesicles include ectosomes, apoptotic bodies, microparticles, microvesicles, and exosomes [66].

**Ectosomes**

A distinct subtype of EVs that ubiquitously sheds from the plasma membrane.

In comparison to exosomes, these vesicles are more variable in size (100–500 nm).

**Apoptotic bodies**

As another major class of EVs (50–5000 nm) [65], these vesicles are a product of cells undergoing programmed cell death. Increasing evidence suggests that this class of vesicles play a role in immunity surveillance [66].

**Exosomes**

Nano-sized membrane bound vesicles ranging 70–150 nm in diameter, which begin as intraluminal vesicles found in the multivesicular body (MVB). These released vesicles can be delivered to distant or nearby cells and serve as mediators for intercellular communication.

**Exosome cargo**

The delivery cargo contained within exosomes includes proteins, lipids, RNAs, DNAs and other cellular components. The molecular contents can be influenced by different physiological and pathological states of the cell. As intercellular communicators, these cargoes can be exchanged, eliminated and illicit cellular response.

**Autophagy-lysosomal pathway**

An evolutionary conserved process that is activated during stress conditions such as starvation, oxidative stress and other damaging conditions. It involves the bulk degradation of macromolecules or organelles by sending this cargo to the lysosome [67,68].

**Lysosome exocytosis**

A Ca^2+^ regulated process leading to the extracellular release of lysosomal content. Lysosomes release their contents by migrating from the perinuclear region to the cell surface and fuse with the PM. Exosomes are also present in lysosomes and can be released through this cellular trafficking pathway [46,69].

**Autophagosomes**

A double membrane compartment that engulfs cytoplasmic substrates destined for degradation. Once the cargo is sequestered, these specialized organelles fuse and deliver their engulfed contents to lysosomes for degradation [67,68].

**Messenger RNA (mRNA)**

This is a single-stranded RNA molecule that is first transcribed from DNA in the nucleus and then exported to the cytoplasm. This transcript encodes the genetic sequence of a gene which is then translated into protein by ribosome complexes.

**MicroRNA (miRNA)**

First discovered in *Caenorhabditis elegans* [70,71], these are a subclass of noncoding RNAs, with an average length of 22 nucleotides [72]. They bind to the complimentary sequence of mRNA molecules and turn off gene expression. This post-transcriptional regulation of gene expression can be carried out through a variety of mechanisms. miRNA has been identified in exosomes, that may be targeted to other cells and modulate gene expression [72].

**Endocytosis**

A cellular process in which cells internalize materials and nutrients from the outside. This process can be subdivided into four pathways: caveolae, pinocytosis, receptor-mediated endocytosis, and phagocytosis.

**Clathrin-coated**

Clathrin is a self-polymerizing protein composed of three heavy and light chains that form the triskelion cage. Clathrin coating was first discovered around vesicles invaginating from the plasma membrane and this remains the best characterized transport vesicle.

**Early endosome (EE)**

As the first compartment of the endocytic pathway, this organelle is found at the periphery of the cell. EEs are noted as tubule-vesicular structures and serve as the principal sorting station.

**Endosomal membranes**

A collection of intracellular membrane bound organelles that make up the endocytic trafficking network.

**Multivesicular bodies (MVB)**

A specialized member of the endo-lysosomal compartment that matures from EE by the inward budding into the lumen [7].

**Intraluminal vesicles (ILVs)**

These vesicles are precursors to exosomes and arise from the inward budding into the lumen of the MVB [7].

**Endosomal sorting complex required for transport (ESCRT)**

This machinery includes four major complexes ESCRT -0, -I, -II, and -III that form a multicomplex of proteins. Together, these complexes control MVB biogenesis and coordinate the sorting of ubiquitinated membrane proteins to the intraluminal vesicles [73].

**Tunneling nanotubules (TnTs)**

These long membrane nanotubules protrude from the plasma membrane of one cell and make contacts with another distant cell. This novel route in cell-to-cell communication enables transfer of proteins, cellular organelles, miRNAs and viral pathogens including HIV and prions [74].

**Astrocytes**

These are specialized glial cells in the CNS. They are the most numerous cell type and harbor a distinct star shaped morphology. This population of cells perform diverse roles such as axon guidance, synaptic support and modulate the blood-brain barrier permeability [75].

**Cortical neurons**

This class of neurons can be divided into interneurons and projection neurons. Interneurons use GABA as a neurotransmitter while projection neurons utilize glutamate [76].

**Oligodendrocytes**

This is a specialized glial cell similar to astrocytes whose responsibility is to myelinate the CNS axons [77].

**Dynamic Light Scattering**

A technique also known as photon correlation spectroscopy, is used to detect the size distribution profile of vesicles by measuring the intensity fluctuations of scattered light when illuminated with a laser beam. This method uses mathematical models derived from Brownian motion and light scattering theory [78].
